# Assessment of COVID-19 Incidence and Severity Among Recipients of Allogenic Stem Cell Transplant After 1 or 2 mRNA Booster Doses During the Omicron Wave in France

**DOI:** 10.1001/jamanetworkopen.2022.47534

**Published:** 2022-12-19

**Authors:** Valentin Letailleur, Amandine Le Bourgeois, Thierry Guillaume, Maxime Jullien, Marianne Coste-Burel, Marie C. Béné, Patrice Chevallier

**Affiliations:** 1 Hematology Department, Nantes University Hospital, Nantes, France; 2 Institut National de la Santé et de la Recherche Médicale Unité Mixte de Recherche UMR1232, Centre de Recherche en Cancérologie et Immunologie Nantes-Angers, Institut de Recherche en Santé de l’Université de Nantes, University of Nantes, France; 3 Virology Department, Nantes University Hospital, Nantes, France; 4 Hematology Biology Department, Nantes University Hospital, Nantes, France

## Abstract

This cohort study assesses the incidence and severity of COVID-19 among vaccinated recipients of allogenic stem cell transplant in a single center after 1 or 2 messenger RNA booster doses during the Omicron wave in France.

## Introduction

Before COVID-19 messenger RNA (mRNA) vaccines were available, SARS-CoV-2 infection was responsible for up to 21% to 25% of deaths among recipients of allogeneic hematopoietic stem cell transplant (HSCT).^1,2^ This incidence was substantially reduced after 2 successive vaccinations, but the humoral response waned after a few months.^3^ Interest in the efficacy of 1 or 2 booster doses has since been confirmed, with a persistent and notably reduced incidence in severe forms of or deaths due to COVID-19.^4^ Over a median 6 months of follow-up for 141 recipients of allogenic HSCT after a third mRNA vaccine dose, we observed high long-term protection.^5^ In the previous cohort, only 2 patients were diagnosed with SARS-CoV-2 infection and did not require hospitalization (1.4%) and only 1 patient died (0.7%).^5^ Since these results were obtained mainly during the Delta wave in the second half of 2021, here we consider COVID-19 incidence and severity among recipients of allogenic HSCT during the first half of 2022 when Omicron was predominant in France.

## Methods

This single-center observational cohort study was approved by the Nantes University Hospital Institutional Review Board. Informed consent was obtained from all participants. The study followed the STROBE reporting guideline.

We aimed to describe the incidence and severity of SARS-CoV-2 infection during the Omicron wave (January 1 to June 30, 2022) for a cohort of French patients who had undergone allotransplantation and were previously fully vaccinated (defined in this study as 2 mRNA vaccine doses plus 1 mRNA booster). Severity was defined as hospitalization and/or death attributable to SARS-CoV-2 infection. Associations of clinical characteristics with infection were investigated using χ^2^ and Wilcoxon tests with R software (R Foundation for Statistical Computing) via BiostaTGV. *P* < .05 (2-tailed) was considered statistically significant.

## Results

This study included 211 patients (121 men [57.3%] and 90 women [42.7%]), with a median age of 59 years (range, 21-78 years). Cohort characteristics and outcomes are described in the Table and Figure. The Table also presents serology assessments^6^ and results. All 211 patients were fully vaccinated; 68 (32.2%) also received a second booster between July 2021 and May 2022 (Figure). Between January 1 and June 30, 2022, 37 patients (17.5%) had a confirmed SARS-CoV-2 infection. Of these, most (35 [94.6%]) had received their last (third or fourth) vaccine dose before infection and had preinfection serology results available.

**Table.** Characteristics of Recipients of Allogenic Hematopoietic Stem Cell Transplant, Antispike Immunoglobulin G Assessments, and Univariate Analysis Results^a^

Characteristic	Values	*P* value
Total No. of recipients	211 (100)	
Sex		
Male	121 (57.3)	NA
Female	90 (42.7)	
Age (on January 1, 2022), median (range), y	59 (21-78)	NA
Delay graft (January 1, 2022), median (range), d	1424 (236-44 562)	NA
<1 y from graft	5 (2.3)	
1-2 y from graft	48 (22.7)	
>2 y from graft	158 (75.0)	
Disease		
Myeloid	136 (64.5)	NA
Lymphoid	69 (32.7)	
Other	6 (2.8)	
Conditioning		
Myeloablative	53 (25.0)	NA
Reduced intensity	148 (70.0)	
Sequential	10 (5.0)	
Donor type		
Sibling	60 (28.0)	NA
Matched unrelated	84 (40.0)	
Haploidentical	51 (24.0)	
9/10 Mismatch unrelated	9 (4.5)	
Cord blood	7 (3.5)	
Ongoing treatment		
Yes	26 (12.0)	NA
No	185 (88.0)	
Previous SARS-CoV-2 infection (before January 1, 2022)	10 (4.7)	NA
No. and timing of mRNA vaccine boosters		
At least 1 (April 1, 2021, to December 28, 2021)	211 (100)	NA
Only 1 (April 14, 2021, to December 23, 2021)	143 (67.8)	
2 (July 2, 2021, to May 1, 2022)	68 (32.2)	
Antispike IgG assessment		
Yes	204 (97.0)	NA
No	7 (3.0)	
Serologic assays		
Roche S tAb	167 (82.0)	NA
Abbott S IgG	7 (3.5)	
DiaSorin TriS	24 (12.0)	
Atellica	12 (6.0)	
Novalisa	1 (0.5)	
Serology timing		
April 20, 2021, to December 31, 2022	171 (84.0)	NA
January 1, 2022, to May 18, 2022	33 (16.0)	
Before last vaccine	65 (32.0)	
Delay, median (range), d	41 (0-115)	
After last vaccine	139 (68.0)	
Delay, median (range), d	179 (3-280)	
Antispike IgG titer, BAU/mL^b^		
Negative	18 (9.0)	NA
Low (>0.8-250)	22 (11.0)	
High (>250)	164 (80.0)	
SARS-CoV-2 infection (univariate analysis), No./total No. (%)		
After transplantation, y		
<1	1/5 (20.0)	.99
>1	36/206 (17.4)	
No. of boosters received		
1	31/143 (21.6)	.02
2	6/68 (8.8)	
Antispike IgG titer, BAU/mL		
Low (≤250)	11/40 (27.5)	.08
High (>250)	26/164 (15.8)	
Delay from allogenic HSCT, y		
<2	27/158 (17.0)	.76
≥2	10/53 (19.0)	
Ongoing immunosuppressive/chemotherapy treatment		
Yes	4/26 (15.3)	.75
No	33/185 (18.0)	
Previous COVID-19 infection	0/10	

Abbreviations: BAU, binding antibody unit; HSCT, hematopoietic stem cell transplant; IgG, immunoglobulin G; mRNA, messenger RNA; NA, not applicable; tAb, total antibody.

^a^ Unless indicated otherwise, data are presented as No. (%) of patients.

^b^ All antibody levels are expressed in BAU per milliliter, which are traceable to the World Health Organization international standard for anti–SARS-CoV-2 immunoglobulin.

**Figure.**
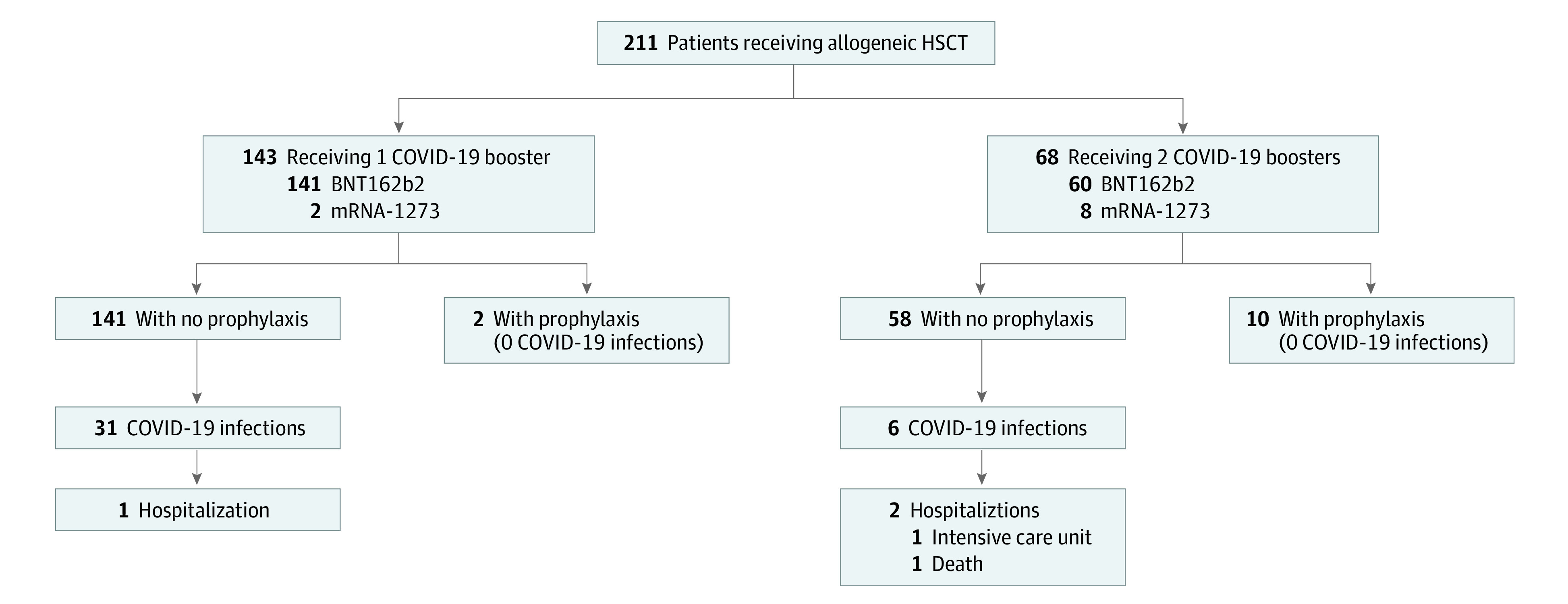
Study Flowchart Describing Vaccine Type and Booster Dose, Prophylaxis, and Clinical Outcomes of Patients Twelve patients had received combination tixagevimab and cilgavimab (Evusheld; AstraZeneca) as anti–COVID-19 prophylaxis because of low immunoglobulin G titer after 1 (n = 2) or 2 (n = 10) boosters. HSCT indicates hematopoietic stem cell transplant.

The SARS-CoV-2 infection rate observed here was significantly higher compared with that for July 1 to December 31, 2021 (17.5% vs 2.1%; *P* < .001), suggesting potentially higher contagiousness of Omicron vs Delta. We noted that 34 of 37 infections (92.0%) were mild; only 3 (1.4%) hospitalizations (including 1 in the intensive care unit [ICU]) and 1 death (0.5%) occurred. The patient hospitalized in the ICU presented with a second mild SARS-CoV-2 infection 2.5 months after the first. Fewer infections were observed for individuals who received a second booster (Table).

## Discussion

The results of this cohort study suggest that during the Omicron wave in the first half of 2022 in France, the incidence of SARS-CoV-2 infection increased significantly among recipients of allogenic HSCT although they were fully vaccinated. We observed that 92.0% of these infections were mild, as only 3 hospitalizations and 1 death were documented in our series. These findings suggest that antispike mRNA vaccines remain effective against Omicron.

Our study has some limitations. The timing of booster doses was not homogeneous for all patients, and characterization of virus variants was not performed. Also, we acknowledge the absence of both assays for neutralizing antibodies and assessments of B-cell memory and T-cell functional response.

Our findings suggest that the significant vaccine protection against severe forms of COVID-19 may be associated with reactivation of memory immune responses upon SARS-CoV-2 infection. Indeed, 1 year after allotransplant, most patients were well protected after full vaccination. However, these observations suggest that a second booster may provide additional protection and should be proposed to patients who receive allotransplant.

## Notes

Supplement.
Data Sharing Statement
