# Supplementary material for: Assessment of COVID-19 Incidence and Severity Among Recipients of Allogenic Stem Cell Transplant After 1 or 2 mRNA Booster Doses During the Omicron Wave in France
Source: JAMA Netw Open. 2022 Dec 19;5(12):e2247534. doi: 10.1001/jamanetworkopen.2022.47534 (PMC9856420; doi:10.1001/jamanetworkopen.2022.47534)
Supplement: Supplement. — Data Sharing Statement [file jamanetwopen-e2247534-s001.pdf]

Data Sharing Statement

Letailleur. Assessment of COVID-19 Incidence and Severity Among Recipients of Allogenic Stem Cell Transplant After 1 or 2 mRNA Booster Doses During the Omicron Wave in France. *JAMA Netw Open*. Published December 19, 2022. doi:10.1001/jamanetworkopen.2022.47534

Data
Data available: No
